# Perceptions and preferences of teaching methods among medical students in Abubakar Tafawa Balewa University, Bauchi, Nigeria – A mixed methods study

**DOI:** 10.1371/journal.pone.0358966

**Published:** 2026-09-23

**Authors:** Solomon Daniel Halilu, Afisulahi Abiodun Maiyegun, Godiya Ishaya Dare, Ganaka Kubi Musa, Yakubu Bababa Shirama, Hassana Dauda Gololo, Appolos Kanda Gizaki

**Affiliations:** 1 Department of Radiology, College of Medical Sciences, Abubakar Tafawa Balewa University, Bauchi, Nigeria; 2 Department of Radiology, Abubakar Tafawa Balewa University Teaching Hospital, Bauchi, Nigeria; 3 Usher Institute, College of Medicine and Veterinary Medicine, University of Edinburgh, Edinburgh BioQuarter, Edinburgh, United Kingdom; 4 Department of Clinical Pharmacology and Therapeutics, College of Medical Sciences, Abubakar Tafawa Balewa University, Bauchi, Nigeria; 5 Department of Family Medicine, Abubakar Tafawa Balewa University Teaching Hospital, Bauchi, Nigeria; 6 Department of Internal Medicine, College of Medical Sciences, Abubakar Tafawa Balewa University, Bauchi, Nigeria; 7 Department of Internal Medicine, Abubakar Tafawa Balewa University Teaching Hospital, Bauchi, Nigeria; 8 Department of Public Health, College of Medical Sciences, Abubakar Tafawa Balewa University, Bauchi, Nigeria; 9 Department of Anaesthesia, Abubakar Tafawa Balewa University Teaching Hospital, Bauchi, Nigeria; 10 Department of Educational Foundations, Abubakar Tafawa Balewa University, Bauchi, Nigeria; Federal University Otuoke, NIGERIA

## Abstract

**Introduction:**

This study investigated the perceptions and preferences of medical students at Abubakar Tafawa Balewa University (ATBU) regarding various teaching methods, including lectures, problem-based learning (PBL), team-based learning (TBL), practical demonstrations, and digital learning tools.

**Methods:**

A cross-sectional survey of 171 medical students was conducted, using a semi-structured questionnaire. Students were selected using stratified random sampling techniques. Quantitative data were analyzed using descriptive statistics (frequencies, percentages) and inferential statistics (Chi-square test) and qualitative data were analysed using thematic content analysis. The thematic content analysis was based on 171 responses.

**Results:**

Practical demonstrations (32.2%) and small group activities (24.0%) were the preferred teaching methods. Key challenges identified include difficulty retaining information (48.0%) and inadequate preparation for clinical practice (15.8%). The students demanded practical, clinically relevant learning, and lecturers who are well-grounded in their subjects and had good teaching skills. Specifically, they preferred lecturers who are medical consultants and could also mentor them.

**Conclusion:**

The teaching curriculum in the school should incorporate more clinically relevant, practical demonstrations with adequate clinical exposure, more group-based learning, and improved digital infrastructure. Retention techniques should be routinely taught to improve learning retention. Finally, more doctors should be employed for training and mentoring the students. Lecturers should be trained in positive teaching and mentoring skills and to avoid student intimidation.

## Introduction

Learning is an interactive and collaborative process where students and teachers partner to facilitate knowledge sharing [1]. In medical education, effective teaching strategies are of paramount importance, not only for knowledge transfer but also for the development of skills crucial for clinical practice. Traditional didactic, subject-centered teaching has increasingly given way to interactive, problem-based learning [2]. While lectures remain a cornerstone and may be more comfortable for some students and lecturers, they are often criticized for being passive and reducing student engagement [3]. On the other hand, student-centered approaches like problem-based learning (PBL) foster critical thinking, an essential skill for successful, independent medical practice [4]. Identifying teaching methods that meet diverse learning needs remains a challenge for many instructors [5].

Alternative learning methods that challenge the traditional methods include problem-based learning (PBL), team-based learning, and digital tools [6,7]. Problem-based learning (PBL) focuses on real-life clinical problems to encourage critical thinking [8]. It is often preferred for its relevance to clinical practice [9]. Virtual simulations allow students to practice clinical skills in a safe, controlled environment without compromising patient safety [10]. Small group activities (SGA) are essential for teamwork development and deeper understanding [1]. Practical demonstrations are preferred for aligning with the practical nature of medical training [5]. Digital learning offers flexibility and accessibility, particularly highlighted during the COVID-19 pandemic and multimodal learning involves visual, auditory, and kinesthetic components to aid learning, thereby meeting the learning needs of visual and other kinds of learners [11,12].

At Abubakar Tafawa Balewa University (ATBU), Bauchi, the medical curriculum employs a combination of these methods, yet little is known about how students perceive them or how their preferences influence academic performance. A lack of alignment between teaching methods and student preferences can lead to lower satisfaction and inadequate preparation for clinical practice.

This study aims to assess the perceptions and preferences of ATBU medical students to identify strategies that better align with their educational needs.

## Methods

### Study site

The study was conducted at the College of Medicine, Abubakar Tafawa Balewa University, Bauchi, Bauchi State, in northeast Nigeria, from 24 January 2025–31 January 2025.

### Population and sampling

The study population comprised medical students enrolled in ATBU’s medical program, divided into two main groups: preclinical students (100 Level–300 Level) and clinical students (400 Level–600 Level). As the 600L students had just graduated while the 100L were in the process of registration, these two classes were excluded from the study. Students from all other classes were included in the study. A total of 171 students were surveyed, with 85 preclinical and 86 clinical students selected to ensure equal representation of both groups.

### Sample and sampling techniques

The sample size was obtained using the Yamane’s formula (1967)


$$\begin{array}{c} n=\frac{N}{\text{1}+N\;{\left(e\right)}^{\text{2}}} \end{array}$$

Where n = Sample size

N = the finite population

e= level of significance (or limit of tolerable error)

1= Unite (a constant value)

There were 283 pre-clinical and clinical medical students.

Substituting for the Sample size using Yamane’s formula:


$$\begin{array}{c} n=\frac{N}{\text{1}+N\;{\left(e\right)}^{\text{2}}} \end{array}$$

$$\begin{array}{c} n=\frac{\text{2}\text{8}\text{3}}{\text{1}+\text{2}\text{8}\text{3}\;{\left(\text{0}.\text{0}\text{5}\right)}^{\text{2}}} \end{array}$$

$$\begin{array}{c} n=\frac{\text{2}\text{8}\text{3}}{\text{1}+(\text{2}\text{8}\text{3}\;x\;\text{0}.\text{0}\text{0}\text{2}\text{5})} \end{array}$$

$$\begin{array}{c} n=\frac{\text{2}\text{8}\text{3}}{\text{1}+\;\text{0}.\text{7}\text{0}\text{7}\text{5}} \end{array}$$

$$\begin{array}{c} n\;=\;165.7=\;166 \end{array}$$

Adding 5 for attrition =166+ 5 =171.

A proportional stratified random sampling technique was used to select participants from each group, ensuring that the sample is representative of each level within both preclinical and clinical stages as shown in Table 1 below:

**Table 1:** Table of selection of participants from each level.

Medical students level	Number in class (n)	Percentage ([n/283]x100)%	Sample (Percentage x 171)
500L	66	23.3	40
400L	76	26.9	46
300L	66	23.3	40
200L	75	26.5	45
Total	283	100	171

### Instrument for data collection

A self-administered semi-structured questionnaire titled “Perception and Preference of Teaching Methods among Medical Students in Abubakar Tafawa Balewa University” was used to elicit information from the respondents. The instrument was divided into two sections: section one (which captured demographic data) and section two (which contained items to be responded to by the respondents).

### Validity of the instrument

To ensure content validity of the instrument, the questionnaire was reviewed by two experts in medical education and curriculum development, and one from measurement and evaluation in the Department of Education Foundation, Faculty of Technology Education, Abubakar Tafawa Balewa University.

### Reliability of the instrument

A pilot study was conducted with 20 students (10 from preclinical and 10 from clinical stages) to assess the clarity, relevance, and reliability (Table 2) of the questionnaire items. Based on feedback from the pilot, necessary adjustments were made to improve the instrument’s comprehensiveness and clarity.

**Table 2:** Reliability.

Variable/Construct	Number of Items	Cronbach Alpha	Internal Consistency status
Effectiveness rating of teaching methods	5	0.79	Reliable
Perceptions of teaching methods	5	0.84	Reliable

The internal consistency of the measurement scales was assessed using Cronbach’s alpha, with a threshold of 0.70 established as the benchmark for acceptable reliability [13]. As presented in Table 2, the subscale for effectiveness rating of teaching methods and perceptions of teaching methods demonstrated acceptable internal consistency with a Cronbach’s alpha of 0.79 and 0.84 respectively, confirming that these items reliably measure the constructs.

### Method for data collection

The questionnaires were distributed in hard copy to students in their lecture venues and they were given adequate time to complete the questionnaire independently, after which the filled questionnaires were retrieved. Data for the qualitative section were collected from the section of the questionnaire where the students were asked about their suggestions for improvement in their studies. The prompt was: “What improvements will you suggest to enhance the teaching methods in your medical school?” The responses were written in free text in the space provided in the questionnaire.

### Method of data analysis

Study Design and Integration: This study employed a convergent, parallel, mixed-methods design (QUAN-qual). Quantitative and qualitative data were collected concurrently in the same questionnaire. The study prioritized quantitative data with an embedded qualitative component. The quantitative phase measured the frequency of effectiveness ratings of teaching methods, learning preferences, satisfaction levels, and challenges across training cadres. The qualitative phase explained why certain methods were preferred and the underlying infrastructure and pedagogical drivers behind identified challenges, while providing suggestions for improvement from students. Data integration was done during the interpretation and synthesis stage.

Quantitative analysis: The collected data were entered into the statistical software Statistical Package for the Social Sciences (SPSS) version 24 for analysis. Descriptive statistics such as frequencies and percentages were calculated to describe the perceptions and preferences of students regarding teaching methods. The data was grouped into preclinical years (200 and 300 levels) and clinical years (400 and 500 levels) and Chi square was used to establish the association between the level of training in medical school (preclinical versus clinical) and other variables. When >20% of the cells in the table have expected count less than 5, Fisher’s exact test is reported instead of Chi square [14]. Missing responses were excluded from analysis. The total values in the tables are reported to the nearest whole number for uniformity. Statistical significance was set at a level of P < 0.05.

Qualitative analysis: Open-ended responses were entered into excel sheet. They were then analyzed thematically using qualitative content analysis. Firstly, two authors, AM and HS, read through the responses and identified the major themes emerging from the qualitative data. Thus, four broad themes were identified: teaching methods, lecturers, teaching facility, and student welfare. The responses were then read several times and coded for until agreement was reached on the final codes, and all comments were placed in appropriate themes. Inter-rater agreement was substantial (Cohen’s Kappa coefficient, 𝜅 = 0.68) [15]. Other co-authors reviewed the original data and the categorization until a consensus was reached. All 171 participants provided responses to the open-ended qualitative question. All responses were analysed.

### Ethical approval

Participants were informed about the study’s purpose and the voluntary nature of their involvement. Informed consent was obtained from all participants, with assurances of confidentiality and anonymity. Informed consent was obtained from students aged 16 and 17 years in line with Section 64(2) of the Child Rights Act [16]. The study was approved by the ethical committee of Abubakar Tafawa Balewa University.

## Results

### Quantitative findings

#### Socio-demographic characteristics.

Table 3 shows the sociodemographic characteristics of the students. The modal age group of the students was 22years to 24 years (40.9%). Respondents were mostly male (65.5%) and from Bauchi State (55%). Although the respondents were fairly evenly distributed between the different levels, the modal levels were 200 level and 400 level (26.3% each).

**Table 3:** Sociodemographic characteristics of the students (n = 171).

Characteristic variable (years)	Frequency	Percent (%)
Age		
16-18	9	5.3
19-21	50	29.2
22-24	70	40.9
25-27	33	19.3
≥28	9	5.3
Total	171	100.0
Gender		
Male	112	65.5
Female	59	34.5
Total	171	100.0
State of Origin		
Adamawa	6	3.5
Anambra	1	0.6
Bauchi	94	55.0
Benue	5	2.9
Borno	1	0.6
Edo	2	1.2
Ekiti	1	0.6
Enugu	2	1.2
Gombe	6	3.5
Jigawa	1	0.6
Kano	6	3.5
Katsina	2	1.2
Kogi	9	5.3
Kwara	5	2.9
Nassarawa	4	2.3
Niger	2	1.2
Ondo	3	1.8
Osun	2	1.2
Oyo	5	2.9
Plateau	6	3.5
Sokoto	1	0.6
Yobe	5	2.9
Zamfara	2	1.2
Total	171	100.0
Religion		
Islam	143	83.6
Christianity	28	16.4
Total	171	100.0
Level of Study		
200L	45	26.3
300L	40	23.4
400L	45	26.3
500L	41	24.0
Total	171	100.0

#### General perceptions of teaching methods.

Table 4 below shows students’ rating of the effectiveness of the teaching methods currently utilized in the College of Medicine, ATBUTH. All methods of teaching were rated highly, with the least cumulative rating of effectiveness (‘effective’ and ‘very effective’) of 52.7% for multimodal/digital Learning). Practical demonstration and lectures were the most preferred methods, with 69.6% and 61.5% of students ranking them as either effective or very effective.

**Table 4:** Effectiveness rating of teaching methods.

Characteristic variable		Number	Percentage (%)
Lecture	Missing variable	10	5.8
	Very ineffective	4	2.3
	Ineffective	11	6.4
	Neutral	41	24.0
	Effective	82	48.0
	Very effective	23	13.5
	Total	171	100.0
Problem-Based Learning	Missing variable	4	2.3
	Very ineffective	3	1.8
	Ineffective	16	9.4
	Neutral	52	30.4
	Effective	61	35.7
	Very effective	35	20.5
	Total	171	100.0
Small group activities/team-based learning	Missing variable	4	2.3
	Very ineffective	7	4.1
	Ineffective	20	11.7
	Neutral	46	26.9
	Effective	61	35.7
	Very effective	33	19.3
	Total	171	100.0
Practical demonstration	Missing variable	3	1.8
	Very ineffective	8	4.7
	Ineffective	17	9.9
	Neutral	24	14.0
	Effective	54	31.6
	Very effective	65	38.0
	Total	171	100.0
Multimodal/Digital Learning	Missing variable	4	2.3
	Very ineffective	6	3.5
	Ineffective	30	17.5
	Neutral	41	24.0
	Effective	48	28.1
	Very effective	42	24.6
	Total	171	100.0

Most students perceived practical demonstrations (32.2%) as the most beneficial teaching method, while multimodal/digital learning was the least preferred (7.6%). Less than half of the students perceived the current teaching methods adequately met their learning needs (“agree” and “strongly agree” = 49.7%) or that the methods helped them in retention of learning (“agree” and “strongly agree” = 39.1%). Similarly, confidence in clinical skills was below 50%, with 3.3% agreeing and 11.7% strongly agreeing that their clinical skills were due to the teaching methods. The remaining data are presented in Table 5 below.

**Table 5:** Students’ perception of teaching methods.

Characteristic variable		Frequency	Percentage (%)
Which of the following teaching methods is most beneficial for your learning needs?	Lectures	30	17.5
	Problem-based learning	31	18.1
	Small group activities (SGA)/Team-based learning	41	24.0
	Practical demonstration	55	32.2
	Multimodal/Digital Learning	13	7.6
	Missing variable	1	0.6
	Total	171	100.0
The current teaching methods used in my program meet my learning needs	Strongly disagree	3	1.8
	Disagree	19	11.1
	Neutral	63	36.8
	Agree	77	45.0
	Strongly agree	8	4.7
	Missing variable	1	0.6
	Total	171	100.0
I’m confident in my clinical skills as a result of the teaching methods	Strongly disagree	2	1.2
	Disagree	24	14.0
	Neutral	67	39.2
	Agree	57	33.3
	Strongly agree	20	11.7
	Missing variable	1	0.6
	Total	171	100.0
The variety of teaching methods keeps me engaged in my studies	Strongly disagree	1	0.6
	Disagree	19	11.1
	Neutral	51	29.8
	Agree	75	43.9
	Strongly agree	25	14.6
	Total	171	100.0
The teaching methods used help me retain information effectively	Strongly disagree	5	2.9
	Disagree	31	18.1
	Neutral	66	38.6
	Agree	56	32.7
	Strongly agree	11	6.4
	Missing variable	2	1.2
	Total	171	100.0

As shown in Table 6, the greatest challenge students face with the current teaching methods was retention of information (48.0%).

**Table 6:** Challenges faced with current teaching methods.

Characteristic variable	Frequency	Percentage (%)
Difficulty in retaining information	82	48.0
Inadequate preparation for clinical practice	27	15.8
Limited interaction or feedback from instructors	24	14.0
Lack of engagement with the content	22	12.9
Insufficient access to practical resources or clinical sessions	12	7.0
Other	1	0.6
Missing variable	3	1.8
Total	171	100.0.

Table 7 below shows students’ perceptions of the importance of incorporating digital learning tools and technologies into the curriculum. Most students (81.3%) believed this was at least very important.

**Table 7:** Importance of incorporating digital learning tools and technologies into the curriculum.

Characteristic variable	Frequency	Percentage (%)
Extremely important	77	45.0
Very important	62	36.3
Moderately important	29	17.0
Missing variable	3	1.8
Total	171	100.0

Most of the students (53.2%) expressed satisfaction with the current teaching methods in the medical school (Table 8). However, over a third of them were neutral.

**Table 8:** Students’ satisfaction with teaching methods.

Characteristic variable	Frequency	Percent (%)
Very satisfied	8	4.7
Satisfied	83	48.5
Neutral	65	38.0
Dissatisfied	12	7.0
Missing variable	3	1.8
Total	171	100.0

Students’ address, age, and religion were significantly associated with their level of medical training (Table 9).

**Table 9:** Association between students’ sociodemographic characteristics and their level of training.

Variable	Clinical	Preclinical	Total	df	Chi Square	P value
Address						
Bauchi	60 (63.8%)	34 (36.2%)	94 (100%)	1	15.30	<0.001
Other States	26 (33.8%)	51 (66.2%)	77 (100%)			
Age (years)				4	77.84^a^	<0.001
16-18	0 (0.0%)	9 (100.0%)	9 (100.0%)			
19- 21	4 (8.0%)	46 (92.0%)	50 (100.0%)			
22-24	48 (68.6%)	22 (31.4%)	70 (100.0%)			
25-27	28 (84.8%)	5 (15.2%)	33 (100.0%)			
28 and above	6 (66.7%)	3 (33.3%)	9 (100.0%)			
Total	86 (50.3%)	85 (49.7%)	171 (100.0%)			
Gender				1	0.74	0.39
Male	59 (52.7%)	53 (47.3%)	112 (100.0%)			
Female	27 (45.8%)	32 (54.2%)	59 (100.0%)			
Total	86 (50.3%)	85 (49.7%)	171 (100.0%)			
Religion				1	8.57	0.003
Islam	79 (55.2%)	64 (44.8%)	143 (100.0%)			
Christianity	7 (25.0%)	21 (75.0%)	28 (100.0%)			

^a^Fisher’s Exact Test was calculated because > 20% of cells had expected count <5.

Preclinical students were more likely to rate Multimodal/Digital Learning as very effective (Table 10). This finding was statistically significant.

**Table 10:** Association between rating of effectiveness of teaching methods and clinical status of students.

Method		Clinical	Preclinical	Total	df	Chi Square	P value
Lecture					4	2.18	0.70
	Very Ineffective	3 (75.0%)	1 (25.0%	4 (100.0%)			
	Ineffective	5 (45.5%)	6 (54.5%)	11 (100.0%)			
	Neutral	18 (43.9%)	23 (56.1%)	41 (100.0%)			
	Effective	44 (53.7%)	38 (46.3%)	82 (100.0%)			
	Very Effective	11 (47.8%)	12 (52.2%)	23 (100.0%)			
	Total	81 (50.3%)	80 (49.7%)	161 (100.0%)			
PBL					4	7.20	0.13
	Very ineffective	3 (100.0%)	0 (0.0%)	3 (100.0%)			
	Ineffective	10 (62.5%)	6 (37.5%)	16 (100.0%)			
	Neutral	21 (40.4%)	31 (59.6%)	52 (100.0%)			
	Effective	31	30	61			
		50.8%	49.2%	100.0%			
	Very Effective	21 (60.0%)	14 (40.0%)	35 (100.0%)			
	Total	86 (51.5%)	81 (48.5%)	167 (100.0%)			
SGA/TBL					4	8.90	0.06
	Very Ineffective	3 (42.9%)	4 (57.1%)	7 (100.0%)			
	Ineffective	15 (75.0%)	5 (25.0%)	20 (100.0%)			
	Neutral	18 (39.1%)	28 (60.9%)	46 (100.0%)			
	Effective	29 (47.5%)	32 (52.5%)	61 (100.0%)			
	Very Effective	20 (60.6%)	13 (39.4%)	33 (100.0%)			
	Total	85 (50.9%)	82 (49.1%)	167 (100.0%)			
Practical Demonstration					4	4.60	0.33
	Very Ineffective	6 (75.0%)	2 (25.0%)	8 (100.0%)			
	Ineffective	11 (64.7%)	6 (35.3%)	17 (100.0%)			
	Neutral	11 (45.8%)	13 (54.2%)	24 (100.0%)			
	Effective	29 (53.7%)	25 (46.3%)	54 (100.0%)			
	Very Effective	29 (44.6%)	36 (55.4%)	65 (100.0%)			
	Total	86 (51.2%)	82 (48.8%)	168 (100.0%)			
Multimodal/Digital Learning					4	14.75	0.005
	Very Ineffective	3 (50.0%)	3 (50.0%)	6 (100.0%)			
	Ineffective	22 (73.3%)	8 (26.7%)	30 (100.0%)			
	Neutral	22 (53.7%)	19 (46.3%)	41 (100.0%)			
	Effective	26 (54.2%)	22 (45.8%)	48 (100.0%)			
	Very Effective	12 (28.6%)	30 (71.4%)	42 (100.0%)			
	Total	85 (50.9%)	82 (49.1%)	167 (100.0%)			

Students’ perceptions of teaching methods were not significantly associated with their level of medical training (Table 11).

**Table 11:** Association between students’ perceptions of teaching methods and clinical status.

	Method	Clinical	Preclinical	Total	df	Chi Square	P value
Which of the following teaching methods is most beneficial for your learning needs?	Lectures	16 (53.3%)	14 (46.7%)	30 (100.0%)	4	5.67	0.23
	PBL	19 (61.3%)	12 (38.7%)	31 (100.0%)			
	SGA/TBL	21 (51.2%)	20 (48.8%)	41 (100.0%)			
	Practical Demonstration	26 (47.3%)	29 (52.7%)	55 (100.0%)			
	Multimodal/Digital Learning	3 (23.1%)	10 (76.9%)	13 (100.0%)			
	Total	85 (50.0%)	85 (50.0%)	170 (100.0%)			
							
The current teaching methods used in the program meet my learning needs	Strongly disagree	2 (66.7%)	1 (33.3%)	3 (100.0%)	4	3.99^a^	0.41
	Disagree	9 (47.4%)	10 (52.6%)	19 (100.0%)			
	Neutral	36 (57.1%)	27 (42.9%)	63 (100.0%)			
	Agree	36 (46.8%)	41 (53.2%)	77 (100.0%)			
	Strongly agree	2 (25.0%)	6 (75.0%)	8 (100.0%)			
	Total	85 (50.0%)	85 (50.0%)	170 (100.0%)			
							
I’m confident in my clinical skills as a result of the teaching methods	Strongly disagree	1 (50.0%)	1 (50.0%)	2 (100.0%)	4	5.12	0.28
	Disagree	13 (54.2%)	11 (45.8%)	24 (100.0%)			
	Neutral	29 (43.3%)	38 (56.7%)	67 (100.0%)			
	Agree	35 (61.4%)	22 (38.6%)	57 (100.0%)			
	Strongly agree	8 (40.0%)	12 (60.0%)	20 (100.0%)			
	Total	86 (50.6%)	84 (49.4%)	170 (100.0%)			
The variety of teaching methods keeps me engaged in my studies	Strongly disagree	1 (100.0%)	0 (0.0%)	1 (100.0%)	4	5.81	0.21
	Disagree	11 (57.9%)	8 (42.1%)	19 (100.0%)			
	Neutral	31 (60.8%)	20 (39.2%)	51 (100.0%)			
	Agree	32 (42.7%)	43 (57.3%)	75 (100.0%)			
	Strongly agree	11 (44.0%)	14 (56.0%)	25 (100.0%)			
	Total	86 (50.3%)	85 (49.7%)	171 (100.0%)			
The teaching methods used help me retain information effectively	Strongly disagree	3 (60.0%)	2 (40.0%)	5 (100.0%)	4	7.98	0.09
	Disagree	22 (71.0%)	9 (29.0%)	31 (100.0%)			
	Neutral	28 (42.4%)	38 (57.6%)	66 (100.0%)			
	Agree	28 (50.0%)	28 (50.0%)	56 (100.0%)			
	Strongly agree	4 (36.4%)	7 (63.6%)	11 (100.0%)			
	Total	85 (50.3%)	84 (49.7%)	169 (100.0%)			

^a^Fisher’s Exact Test was calculated because > 20% of cells had expected count <5.

There was no statistically significant association between students’ perception of the importance of digital learning tools and their level of training (Table 12).

**Table 12:** Association between students’ perception of the importance of incorporating digital tools and their preclinical/clinical status.

		Clinical	Preclinical	Total	df	Chi square	P value
Importance of incorporating digital learning tools into the curriculum	Extremely important	35 (45.5%)	42 (54.5%)	77 (100.0%)	2	2.33	0.31
	Very important	31 (50.0%)	31 (50.0%)	62 (100.0%)			
	Moderately important	18 (62.1%)	11 (37.9%)	29 (100.0%)			
	Total	84 (50.0%)	84 (50.0%)	168 (100.0%)			

Students in clinical years were more likely to assess the interaction and feedback from instructors as inadequate, while more of the students in preclinical classes reported that the current teaching methods did not prepare them well for clinical practice (Table 13). This finding was statistically significant.

**Table 13:** Challenges faced with current teaching methods.

Challenges	Clinical	Preclinical	Total	df	Chi Square	P value
Difficulty in retaining information	31 (37.8%)	51 (62.2%)	82 (100.0%)	5	13.63	0.02
Inadequate preparation for clinical practice	20 (74.1%)	7 (25.9%)	27 (100.0%)			
Limited interaction or feedback from instructors	12 (50.0%)	12 (50.0%)	24 (100.0%)			
Lack of engagement with the content	12 (54.5%)	10 (45.5%)	22 (100.0%)			
Insufficient access to practical resources or clinical sessions	8 (66.7%)	4 (33.3%)	12 (100.0%)			
Other	0 (0.0%)	1 (100.0%)	1 (100.0%)			
Total	83 (49.4%)	85 (50.6%)	168 (100.0%)			

There was no statistically significant difference in overall satisfaction with teaching methods between students in the clinical years and those in the preclinical years (Table 14).

**Table 14:** Overall satisfaction with teaching methods.

Level of Satisfaction	Clinical	Preclinical	Total	df	Chi Square	P value
Very satisfied	4 (50.0%)	4 (50.0%)	8 (100.0%)	3	1.51^a^	0.71
Satisfied	41 (49.4%)	42 (50.6%)	83 (100.0%)			
Neutral	31 (47.7%)	34 (52.3%)	65 (100.0%)			
Dissatisfied	8 (66.7%)	4 (33.3%)	12 (100.0%)			
Total	84 (50.0%)	84 (50.0%)	168 (100.0%)			

^a^Fisher’s Exact Test was calculated because > 20% of cells had expected value <5.

### Qualitative findings

Table 15 below displays the main themes and the codes that emerged from the study.

**Table 15:** Main themes and codes of the study.

Themes	Codes
Teaching methods	‘practical experience’, ‘Simulation and digital’, ’clinical relevance’, ‘learning bite and pace’, ‘group discussion and projects’
Lecturers	‘Increase staff strength,’ ‘Relevant staff specialty,’ ‘Teaching skills and competence,’ ‘Mentoring and guidance’
Facility	‘Physical structures,’ ‘Digital & audiovisual structures’, ‘Practical tools/resources’
Student Welfare	‘Infrastructure,’ ‘Lecturers’ attitude’

Most students were concerned about teaching methods which provided practical experience and were clinically relevant (Table 16). They also wanted interactive lecturers with in-depth knowledge of the subjects, who could mentor, and preferably were medical consultants (Table 17). They opined for increasing the staff strength to improve teaching efficiency. The students laid approximately equal emphasis on facilities such as practical tools, physical structures, and digital and audiovisual resources (Table 18). Some students raised concerns about their welfare, which included improving their living and study conditions as well as addressing cohesive and threatening attitudes of some lecturers, which could harm their mental health (Table 19).

**Table 16:** Theme: Teaching methods.

Codes	Frequency	Representative quote
Practical experience	23	More practical learning and less theoretical contents, sometimes theory is overwhelming and unnecessary
Clinically relevant	17	Engagement in activities that [is] relevant to clinical practice; more emphasis should be made on some clinically related topics in the preclinical, more emphasis on acquiring the clinical skills rather than to just pass exams …
Simulation and digital	9	… adopt digital teaching methods
Learning bite and pace	7	They should not overbombard us, they should only take the relevant training; let the training be slow and steady, no need to rush
Group discussion and projects	5	Group projects and presentations; small group discussion should be encouraged
Total	61	

**Table 17:** Theme: Lecturers.

Codes	Frequency	Representative quote
Teaching skills and competence	17	Make lectures interesting and simplify it; There should be more time to interact with the instructors; some of the lecturers should have good and deep understanding of what they are coming to teach instead of just reading their slides out to us; the lecturers should teach rather than lecturing
Mentoring and guidance	9	Encourage students to get goals for improvement and participation; improve interaction with instructors
Increase staff strength	5	(”[sic]” indicates “as written by the respondent”) The school should employ more staff as the little [sic] we have most of them get overwhelmed and tired [and] often and often don’t have time for students
Relevant staff specialty	2	Provision of more specialist[s] to help in the program; More clinical exposure and contacts with consultants; try to bring medical doctors to be lecturers in our college
Total	33	

**Table 18:** Theme: Facility.

Code	Frequency	Representative Quote
Practical tools/resources	9	More practical equipment and tools improve understanding; There should be more practical demonstrations and projection.
Physical structures	8	Use of comfortable chairs; Provision of the latest medical books in the library; Provide a good place for taking lectures; There should be adequate fresh cadavers for proper understanding of gross anatomy
Digital & audiovisual structures	8	I suggest having more advance digital learning materials; Provision of effective digital computers for practical demonstrations of internal organs/systems.
Total	25	

**Table 19:** Theme: Students’ welfare.

Code	Frequency	Representative Quote
Infrastructure	4	I suggest that the university should provide generator and Wi-Fi to the college; the university should please try and improve the welfarism of the students, particularly those in Gubi Campus.
Lecturers’ attitude	2	Bullying medical students should be discouraged because of their mental health; I think less crowd will help me focus. Less intimidation from the lectures too.
Total	6	

## Discussion

### Sociodemographic characteristics of the students

This study reported the perception of medical students of ATBUTH, in Bauchi, northeast Nigeria about the teaching methods employed in the medical programme. Male students were nearly twice as many as female students, reflecting the long-standing tradition of gender imbalance in medical colleges and institutions [17,18]. This calls for action to correct the imbalance and encourage female representation in medical schools in Nigeria.

About 60% of the students were within the ages of 22 years to 27 years, despite 16 years to 17 years being the age at secondary school graduation in Nigeria. In a smooth transition from secondary to university education, students in the fifth year of university would usually be between the age range of 22 years to 23 years. The advanced age of the students in this study, therefore, reflects the many years many students spend while attempting to secure placement in the competitive medical schools after secondary school education. On the positive side, the age range implies that students in this medical school are adults with supposed learning skills appropriate for the rigors of advanced medical education.

Most students were from the northern states of the country. This is most likely because of the catchment area policy of federal universities in Nigeria, which ensures that a quota of students are enrolled from each university’s geopolitical environment [19,20]. Another possible reason is the high level of insecurity associated with bandits and terrorist organisations more active in the north, such as Boko Haram and the Islamic State West Africa Province (ISWAP) [21,22].

### Learning preferences

This study highlights a clear preference among ATBU medical students for practical demonstrations (32.2%) and small group activities (SGA/TBL) (24%) over traditional didactic lectures. This shift mirrors global trends in medical education moving from purely didactic to interactive, student-centered models [23,24]. Studies that reported preference of SGA over lectures include Latha et al in India, where 82% of medical students preferred it to didactic lectures [25]. Similarly, Cruz, in the Philippines, reported that 66% of the medical students preferred small group learning and 62% preferred hands-on training [26]. These studies contrast with the findings of some earlier researchers such as Ismail et al in Malaysia where 72% of the students preferred didactic lecture [27].

Interestingly, while 81.3% of students recognized the importance of digital tools, only 7.6% identified them as their most beneficial method. This utility-preference gap may stem from the qualitative findings where students noted a lack of digital and audiovisual structures and reliable infrastructure like Wi-Fi and electricity. Therefore, despite acknowledging the potential advantages of digital learning tools, they cannot benefit from them due to infrastructural deficiencies. This finding illustrates a significant barrier to learning in low- and middle-income countries like Nigeria [28,29].

The most significant challenge identified was difficulty in retaining information (48%), and this problem was significantly higher in the preclinical years. This suggests that while current methods are perceived as “effective” by over half the cohort, they may not be fostering the deep, long-term learning required for clinical mastery. This may be due to the information overload from largely didactic lectures that some of the students pointed out, in contrast to the more active modes of learning (SGA and practical demonstration) [30,31]. This challenge of retention prevalent in the preclinical years – where the foundation for medical training is formed- probably reflects on learning in the clinical years, as students in clinical years (74.1%) were significantly more likely to report inadequate preparation for clinical practice than preclinical students (25.9%).

This finding argues for a comprehensive review of the medical curriculum to increase practical and interactive components and the need to train students on efficient study habits.

The qualitative data further emphasizes a desire for “clinically relevant” content and “mentoring” from lecturers, suggesting that students feel a disconnect between preclinical theory and bedside practice. This may explain the low confidence students reported in their clinical skills and highlights the importance for doctors as the core teaching staff as they are best positioned to impact clinical knowledge and mentoring to medical trainees [32]. This idea is demonstrated by the students’ demand for “medical doctors,” “consultants,” and “specialists” as lecturers.

Addressing the “threatening attitudes” of some lecturers is also critical, as intimidation is known to hinder the physical and psychological safety necessary for effective learning in medical environments [33]. Although the nature of the perceived threats was not specified in the study, examples of such threats reported in the literature include verbal abuse, mainly by medical consultants and residents, with prevalence ranging between 46% and 91% [34,35]. Given that this abuse is often unreported because of the fear of reprisal, the university must endeavour to provide adequate support to students. In addition, the lecturers should be trained in appropriate non-intimidating communication skills and productive lecturer-student interactions.

### Study strengths and limitations

#### Strengths.

- Mixed-Methods Approach: The integration of quantitative survey data with qualitative thematic analysis provides a comprehensive view of the students’ learning preferences
- Representative Sampling: The use of proportional stratified random sampling ensured a balanced perspective from both preclinical and clinical students.

#### Limitations.

- Single-Center Study: Data from a single university in Northeast Nigeria may not be generalizable to all medical schools in Nigeria or globally.
- Cross-Sectional Design: This design captures a snapshot in time and cannot establish a causal link between teaching methods and actual academic performance.
- Self-Report Bias: The data relies on student perceptions, which may be influenced by social desirability or recent exam experiences rather than objective educational efficacy.
- Multiple chi-square tests (Tables 9-14) were conducted without adjustment. Therefore, some statistically significant findings might be chance findings.

#### Recommendations.

- Increased use of non-didactic methods of lecturing such as practical demonstration and small group activities
- Provision of facilities for digital learning such as stable electricity and internet service
- Increased recruitment and training of medical doctors as teaching staff and mentors

## Conclusion

Medical students at ATBU show a strong preference for interactive, practical, and group-based learning over traditional lectures. While there is high awareness of the importance of digital tools, infrastructure deficits remain a barrier to their effective use. To optimize medical training, the curriculum should pivot toward a blended learning approach that prioritizes clinical relevance, increases staff-to-student interaction, recruits more doctors as lecturers, and improves digital infrastructure. Reducing lecturer intimidation and focusing on information retention strategies are essential for developing confident, clinically competent future physicians.
